# Rhodanineacetic Acid Derivatives as Potential Drugs: Preparation, Hydrophobic Properties and Antifungal Activity of (5-Arylalkylidene-4-oxo-2-thioxo-1,3-thiazolidin-3-yl)acetic Acids ^†^

**DOI:** 10.3390/molecules14104197

**Published:** 2009-10-20

**Authors:** Jan Dolezel, Petra Hirsova, Veronika Opletalova, Jiri Dohnal, Vejsova Marcela, Jiri Kunes, Josef Jampilek

**Affiliations:** 1 Department of Pharmaceutical Chemistry and Drug Control, Faculty of Pharmacy in Hradec Kralove, Charles University in Prague, Heyrovskeho 1203, 500 05 Hradec Kralove, Czech Republic; E-Mails: veronika.opletalova@faf.cuni.cz (V.O.); jan.dolezel@faf.cuni.cz (J.D.); hirsovap@lfhk.cuni.cz (P.H.); 2 Zentiva k.s., U kabelovny 130, 102 37 Prague 10, Czech Republic; E-Mail: jiri.dohnal@zentiva.cz (J.D.); 3 Department of Chemical Drugs, Faculty of Pharmacy, University of Veterinary and Pharmaceutical Sciences, Palackeho 1-3, 612 42 Brno, Czech Republic; 4 Department of Clinical Microbiology, Charles University Medical School and Teaching Hospital, Sokolska 581, Hradec Kralove 500 05, Czech Republic; E-Mail: marcela.vejsova@faf.cuni.cz (M.V.); 5 Department of Inorganic and Organic Chemistry, Faculty of Pharmacy in Hradec Kralove, Charles University in Prague, Heyrovskeho 1203, 500 05 Hradec Kralove, Czech Republic; E-Mail: jiri.kunes@faf.cuni.cz (J.K.)

**Keywords:** [(5*Z*)-(5-arylalkylidene-4-oxo-2-thioxo-1,3-thiazolidin-3-yl)]acetic acids, synthesis, lipophilicity measurement, *in vitro* antifungal activity

## Abstract

Some [(5*Z*)-(5-arylalkylidene-4-oxo-2-thioxo-1,3-thiazolidin-3-yl)]acetic acids were prepared as potential antifungal compounds. The general synthetic approach to all synthesized compounds is presented. Lipophilicity of all the discussed rhodanine-3-acetic acid derivatives was analyzed using a reversed phase high performance liquid chromatography (RP-HPLC) method. The procedure was performed under isocratic conditions with methanol as an organic modifier in the mobile phase using an end-capped non-polar C_18_ stationary RP column. The RP-HPLC retention parameter log *k* (the logarithm of the capacity factor *k*) is compared with log *P* values calculated *in silico*. All compounds were evaluated for antifungal effects against selected fungal species. Most compounds exhibited no interesting activity, and only {(5*Z*)-[4-oxo-5-(pyridin-2-ylmethylidene)-2-thioxo-1,3-thiazolidin-3-yl]}acetic acid strongly inhibited the growth of *Candida tropicalis* 156, *Candida krusei* E 28, *Candida glabrata* 20/I and *Trichosporon asahii* 1188.

## 1. Introduction

In 1997, a study based on a database search showed that the prevalence of rhodanine-containing compounds of pharmaceutical interest is very small, despite the fact that the compounds exhibit a wide variety of bioactivities [1]. One of the reasons may be poor solubility of rhodanine derivatives in water, but in rhodanine-3-acetic acids this problem can be overcome by preparing suitable salts.

**Figure 1:**
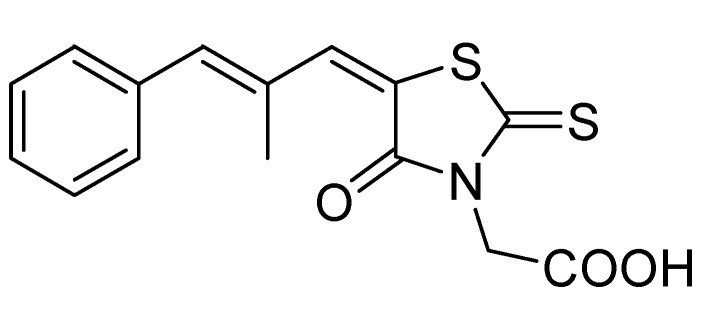
Structure of epalrestat.

Rhodanine-3-acetic acid (RAA) was prepared by Körner [2] in 1908, and condensation products of the acid with various aldehydes were reported in the same year [3]. Since that time, many (5-arylalkylidene-4-oxo-2-thioxo-1,3-thiazolidin-3-yl)alkanoic acids have been prepared and studied as potential antimycobacterial [4,5], antifungal [6,7,8,9,10,11,12,13,14,15], pesticidal [16,17,18], antihypertensive [19], and antineoplastic [20,21] agents. Rhodanine carboxylic acid derivatives have also be patented for the treatment and prevention of metabolic bone disorders. It was found that they stimulate parathyroid hormone receptor-mediated cAMP formation and could be useful for the local and systemic treatment of rheumatoid arthritis, osteoarthritis and degenerative arthrosis [22,23]. Trypanocidal activity of substituted rhodanine-3-acetic acids has been reported recently [24]. The only rhodanineacetic acid derivative that has been used clinically is the aldose reductase inhibitor epalrestat (Figure 1). It is marketed in Japan and used to slow eye damage associated with diabetes and to prevent diabetic peripheral neuropathy [1,25,26,27,28]. Aldose reductase is not the only enzyme inhibited by rhodaninecarboxylic acids. It was found that many other enzymes are inhibited by the derivatives of this structural class, and the enzyme inhibition may be, at least in part, the mechanism responsible for various biological effects of rhodanine derivatives [29].

Determination of the physico-chemical parameters of biologically active compounds has become more important in an age of rational thinking in drug design [30]. One of the major prerequisites for pharmacological screening and drug development is prediction of absorption, e.g., transport of a molecule through cellular membranes, *i.e.* bioavailability. Most frequently, the drugs cross biological barriers by passive transport, which strongly depends on lipophilicity. Therefore hydrophobicity is one of the most important physical properties of biologically active compounds. This thermodynamic parameter describes the partitioning of a compound between an aqueous and an organic phase and is characterized by the partition coefficient *P* [31,32]. For practical purposes, partition coefficient is mostly used in its logarithmic form log *P*. Classical methods for determination of this constant are time consuming and not always sufficiently reliable. Therefore, reversed phase high performance liquid chromatography (RP-HPLC) methods have become popular and are widely used for lipophilicity measurement [33]. This paper is a follow-up work to previous papers [34,35,36,37,38,39,40,41,42,43,44,45,46] aimed at the synthesis, physicochemical properties and biological testing of newly prepared potential drugs based on nitrogen containing heterocycles.

## 2. Results and Discussion

### 2.1. Chemistry

**Scheme 1:**
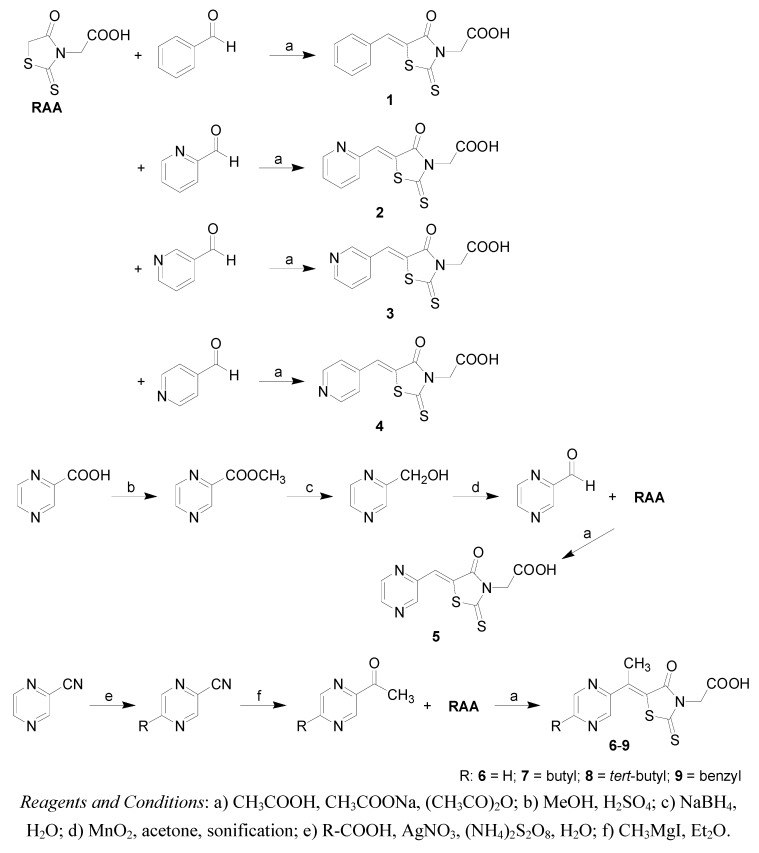
Synthesis and structures of the target 5-substituted rhodanine-3-acetic acid derivatives **1**-**9**.

The preparation of the studied compounds is indicated in Scheme 1. The purity of samples was checked by elemental analysis. Their structures were confirmed by melting points and spectral data (UV, IR, ^1^H- and ^13^C-NMR). Compounds **1**-**4** have been reported previously. [(5*Z*)-(5-Benzylidene-4-oxo-2-thioxo-1,3-thiazolidin-3-yl)]acetic acid (**1**) has been known since 1908 [3], but its NMR characterization was only performed as late as 1982 [27]. In 2006, the compound was prepared under microwave irradiation [47]. The condensation products of rhodanine-3-acetic acid with pyridinecarbaldehydes were prepared in 1961 as potential antibacterial and antifungal agents [8]. {(5*Z*)-[4-oxo-5-(pyridin-2-ylmethylidene)-2-thioxo-1,3-thiazolidin-3-yl]}acetic acid (**2**) was patented as a potential drug for the treatment of metabolic bone diseases [22,23]. The ^1^H-NMR spectra of **3** and **4** were published by Tanaouchi *et al*. [27,28]. The remaining products are novel compounds.

Arylalkylidenerhodanines can form two isomers. According to references [48,49,50,51], syntheses of these compounds result in the thermodynamically more stable *Z-*izomers. Configuration on the exocyclic double bond can be determined on the basis of NMR spectra. ^1^H-NMR signals of the methine-group hydrogens for *Z-*isomers are more downfield compared to *E-*isomers. Calculated and previously reported experimental values of ^1^H-NMR shifts for the products reported in the present paper are given in Table 1. Based on our experimental NMR results the compounds presented here were obtained as single isomers. Their shifts for methine proton range between 7.86-7.94 ppm. Hence, it can be concluded that they are *Z-*isomers.

### 2.2. Lipophilicity

Many low molecular weight drugs cross biological membranes through passive transport, which strongly depends on their lipophilicity. Lipophilicity is a property that has a major effect on absorption, distribution, metabolism, excretion, and toxicity (ADME/Tox) properties as well as pharmacological activity. Lipophilicity has been studied and applied as an important drug property for decades [52].

Log *P* is the logarithm of the partition coefficient in a biphasic system (e.g., *n*-octanol/water), defined as the ratio of compound concentration in both organic/inorganic phases. The log *P* is determined for uncharged species of the drug. Clog *P* values present the logarithm of *n*-octanol/water partition coefficient based on established chemical interactions.

**Table 1:** Lipophilicities and ^1^H-NMR shifts for the methine hydrogen (δ, ppm) of 5-arylalkylidene-3-carboxymethylrhodanines **1**-**9**.

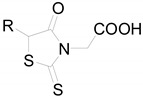						
**Comp.**	**R**	**log *k***	**log *P***	***Z***	***E***	**ND**
**1**	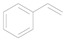	0.2013	2.34 ± 0.81*^a^*	7.41*^b^*	6.80*^b^*	7.88*^c^*7.81*^d^*
			1.54*^b^*			
**2**	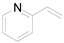	0.1399	0.85 ± 0.82*^a^*	7.62*^b^*	7.01*^b^*	-
			0.62*^b^*			
**3**	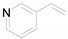	0.1116	1.10 ± 0.82*^a^*	7.41*^b^*	6.80*^b^*	7.95*^c^*
			0.20*^b^*			
**4**	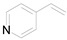	0.1342	0.85 ± 0.82*^a^*	7.39*^b^*	6.78*^b^*	7.87*^c^*
			0.20*^b^*			
**5**	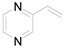	0.1165	0.09 ± 0.82*^a^*	7.41*^b^*	6.80*^b^*	-
			-0.71*^b^*			
**6**	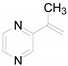	0.1734	0.65 ± 0.84*^a^*	-	-	-
			-0.54*^b^*			
**7**	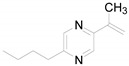	0.2270	2.70 ± 0.84*^a^*	-	-	-
			1.49*^b^*			
**8**	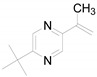	0.2301	2.33 ± 0.84	-	-	-
			*^a^*1.59*^b^*			
**9**	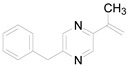	0.2276	2.64 ± 0.84*^a^*	-	-	-
			1.84*^b^*			

*^a^*ACD/LogP, ver. 1.0; *^b^*CS ChemOffice Ultra, ver. 7.0; *^c^*DMSO-*d*_6_, ref. [27,28]; *^d^*DMSO-*d*_6_, ref. [47]; ND = not defined.

It was recognised that the retention of a compound in the reversed-phase liquid chromatography is governed by its lipophilicity, and thus shows correlation with the octanol-water partition coefficient [53]. High performance liquid chromatography (HPLC) provides an excellent platform for computer controlled automated measurements with computerised data acquisition for a large number of compounds. Other advantages in the use of HPLC retention data for lipophilicity determination are the absence of need for concentration determination and method validation, separation of small impurities from the main component, sufficiency of small amounts of material for measurements and possibility of their full automation. Therefore, the investigation of the true potential of this method is of great importance [54].

An excellent review of the effect of stationary and mobile phase selection has been published by van der Waterbeemd *et al*. [32] and more recently by Claessens *et al*. [55]. RP-HPLC methods have become popular and widely used for lipophilicity measurements. A general procedure is the measurement of the directly accessible retention time under isocratic conditions with varying amounts of methanol as an organic modifier in the mobile phase using end-capped non-polar C_18_ stationary RP columns and calculating capacity factors *k*. Log *k* is the logarithm of capacity factors in chromatographic approaches, which is related to the partitioning of a compound between a mobile and a (pseudo-)stationary phase. Log *k* is used as the lipophilicity index converted to log *P* scale [32,33,54,55,56,57].

Some groups used a C_18_ chromatographic column with methanol-water mobile phases to obtain log *k*_w_, *i.e.*, log *k* extrapolated to 0% organic modifier, as an alternative to log *P* [58]. The log *k*_w_ is obtained by performing several measurements with various ratios of water/organic solvent. Nevertheless, determination of log *k*_w_ has some disadvantages. Its determination is time consuming due to a number of measurements before the calculation of log *k*_w_ [53]. Many studies [59,60,61] showed that for heteroaromatic compounds in which various intramolecular interactions between heteroatoms and substituents occur, it is more convenient to determine log *k* using mobile phases containing around 50% methanol. Therefore, this study was performed using methanol/water (70:30) as the mobile phase. The conditions (non-buffered mobile phase) were chosen with respect to conditions of biological evaluations, which are performed mostly under neutral conditions (pH ~7).

**Figure 2:**
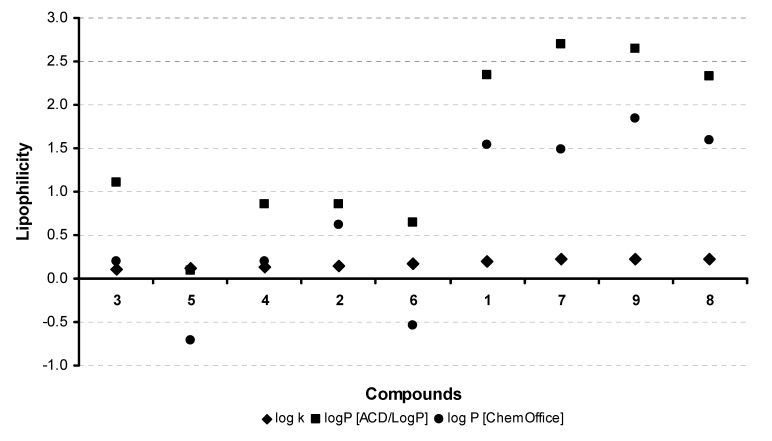
Comparison of the log *P* data calculated using the two programs with the experimentally found log *k* values. The compounds are arranged in the ascending manner according to the experimental log *k* values.

Hydrophobicities (log *P*) of the studied compounds **1**-**9** were calculated using ACD/LogP, version 1.0 (Advanced Chemistry Development Inc., Toronto, ON, Canada) and CS ChemOffice Ultra, version 7.0 (CambridgeSoft, Cambridge MA, U.S.A.) and measured by means of RP-HPLC determination of capacity factors *k* with a subsequent calculation of log *k*. The results are summarized in Table 1 and illustrated in Figure 2.

As expected, methylation of the connection linker increases lipophilicity, *i.e. ,* compound **5** (log *k* = 0.1165) is less hydrophobic than its alkylated derivative **6** (log *k* = 0.1734). Among the arylmethylidene derivatives [(5*Z*)-(5-benzylidene-4-oxo-2-thioxo-1,3-thiazolidin-3-yl)]acetic acid (**1**) is more lipophilic than its 5-heteroarymethylidene congeners **2**-**5**. [(5*Z*)-{5-[1-(5-*tert*-Butylpyrazin-2-yl)ethylidene]-4-oxo-2-thioxo-1,3-thiazolidin-3-yl}]acetic acid (**8**) is the most lipophilic compound, which is in a good agreement with the results of our previous studies [36,37,38,43,44].

### 2.3. In vitro antifungal activity

Antifungal activity of all compounds against *Candida albicans* ATCC 44859 (CA), *Candida tropicalis* 156 (CT), *Candida krusei* E 28 (CK), *Candida glabrata* 20/I (CG), *Trichosporon asahii* 1188 (TA), *Aspergillus fumigatus* 231 (AF), *Absidia corymbifera* 272 (AC) and *Trichophyton mentagrophytes* 445 (TM) was evaluated by the microdilution broth method. These clinical isolates were obtained from the Department of Clinical Microbiology, University Hospital and Faculty of Medicine, Charles University, Prague, Czech Republic. Solubility of all compounds for biological experiments was satisfactory, and the preparation of suitable salts was not necessary. The prepared substances did not show antifungal activity, except for {(5*Z*)-[4-oxo-5-(pyridin-2-ylmethylidene)-2-thioxo-1,3-thiazolidin-3-yl]}acetic acid (**2**), which strongly inhibited the growth of *Candida tropicalis* 156, *Candida krusei* E 28, *Candida glabrata* 20/I and *Trichosporon asahii* 1188. This is rather surprising since Sortino and co-workers [62], who studied antifungal properties of 3**-**unsubstituted 5-arylalkylidenerhodanines, found that the replacement of benzene ring with a pyridine ring resulted in the loss of antifungal activity. Antifungal properties of [(5*Z*)-(5-pyrazin-2-ylalkylidene-4-oxo-2-thioxo-1,3-thiazolidin-3-yl)]acetic acids **5**-**9** have not been studied so far. Similarly to 3-unsubstituted (5*Z*)-5-pyrazine-2-ylalkylidene-2-thioxo-1,3-thiazolidin-4-ones, that have also been prepared and tested in our laboratory [43,63], their corresponding 3-carboxymethyl congeners reported here were also inactive.

**Figure 3:**
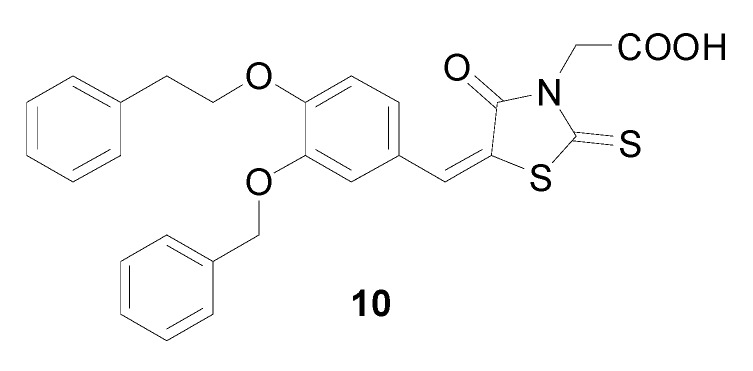
Structure of compound **10**, PMT1 inhibitor.

Little is known about the molecular mechanisms responsible for the antifungal properties of rhodanine-3-acetic derivatives. Orchard *et al*. [13] found that phenylalkoxy substituted (5-benzylidene-4-oxo-2-thioxo-1,3-thiazolidin-3-yl)acetic acids, for example compound **10** (Figure 3), inhibit fungal protein mannosyl transferase 1 (PMT1) and *Candida albicans* proliferation *in vitro*. In some cases, inhibitory activity against the organism outstripped that versus isolated enzyme (PMT1). This indicates an off-target activity (including inhibition of other PMT subtypes) contributing to the anti-*Candida* effects, or increased penetration to the endoplasmic reticulum membrane.

**Table 2:** *In vitro* antifungal activity (IC_80_) of compounds **1**-**9** compared with fluconazole (FLU) standard.

**Strain**		MIC/IC_80_ [μmol/L]									
		1	2	3	4	5	6	7	8	9	FLU
**CA**	*24h*	>500	500	>500	>500	>250	>500	>500	>500	500	1.09
	*48h*	>500	500	>500	>500	>250	>500	>500	>500	>500	2.17
**CT**	*24h*	>500	0.98	500	>500	>250	500	500	>500	>500	2,72
	*48h*	>500	1.95	>500	>500	>250	>500	>500	>500	>500	5.44
**CK**	*24h*	>500	1.95	500	>500	>250	500	500	>500	>500	87.07
	*48h*	>500	1.95	>500	>500	>250	>500	>500	>500	>500	174.14
**CG**	*24h*	>500	0.98	250	>500	>250	250	250	>500	>500	21.77
	*48h*	>500	1.95	500	>500	>250	500	500	>500	>500	69.65
**TA**	*24h*	>500	1.95	500	>500	>250	500	500	>500	>500	4.35
	*48h*	>500	1.95	>500	>500	>250	>500	>500	>500	>500	8.71
**AF**	*24h*	>500	>500	>500	>500	>250	>500	>500	>500	>500	>419
	*48h*	>500	>500	>500	>500	>250	>500	>500	>500	>500	>419
**AC**	*24h*	>500	>500	>500	>500	>250	>500	>500	>500	>500	>128
	48h	>500	>500	>500	>500	>250	>500	>500	>500	>500	>128
**TM**	72h	>500	>500	>500	>500	>250	>500	>500	>500	>500	5.04
	120h	>500	>500	>500	>500	>250	>500	>500	>500	>500	8.00

Unfortunately, the study has not included any simple (5-phenylalkylidene-4-oxo-2-thioxo-1,3-thiazolidin-3-yl)acetic acid lacking phenylalkoxy substituents. Hence, it cannot be concluded whether this type of substitution is necessary for the PMT1-inhibitory activity. Regarding the rhodanineacetic part of the molecule, it was found that the free carboxylic group is needed since the analogues with an ester or amide group did not displayed activity in either the PMT1 or *C. albicans* assays.

## 3. Conclusions

A series of nine [(5*Z*)-(5-arylalkylidene-4-oxo-2-thioxo-1,3-thiazolidin-3-yl)]acetic acids was prepared. Four of the compounds have been reported previously, and five are novel compounds reported in the present paper for the first time. Antifungal properties of the studied compounds against a standard panel of eight fungal strains were tested. Only one compound, {(5*Z*)-[4-oxo-5-(pyridin-2-ylmethylidene)-2-thioxo-1,3-thiazolidin-3-yl]}acetic acid (**2**), inhibited the growth of *Candida tropicalis* 156, *Candida krusei* E 28, *Candida glabrata* 20/I and *Trichosporon asahii* 1188. The remaining substances were inactive. Further studies are required to elucidate the mechanism of antifungal effects of (5-arylalkylidene-4-oxo-2-thioxo-1,3-thiazolidin-3-yl)acetic acids and formulate structure-activity relationships.

## 4. Experimental

### 4.1. General

The synthesis of pyrazinecarbaldehyde and 5-substituted acetylpyrazines was described previously by Opletalova *et al*. [31,32,41]. Commercially available rhodanine-*N*-acetic acid (Fluka), benzaldehyde (VEB Laborchemie), pyridine-2-carbaldehyde (Aldrich), pyridine-3-carbaldehyde (Aldrich), pyridine-4-carbaldehyde (Aldrich), acetophenone (Reachim) were used for condensation. Pyrazinecarbonitrile (Fluka), pyrazinecarboxylic acid (Aldrich) were used as starting materials. For analysis, the samples of compounds were dried 24 hours in the dessicator at 1.33 kPa. The melting points were determined on a Boetius PHMK 05 (VEB Kombinat Nagema, Radebeul, Germany) and are uncorrected. Elemental analyses were performed with an EA 1110 CHNS Analyzer (Carlo Erba). The purity of the final compounds was checked by the HPLC separation module Waters Alliance 2695 XE (Waters Corp., Milford, MA, U.S.A.). The detection wavelength 210 nm was chosen. The solvent peaks in the (blank) chromatogram were deducted from the peaks in the chromatogram of the sample solution. The purity of individual compounds was determined from the area peaks in the chromatogram of the sample solution. UV spectra (λ, nm) were determined on a Waters Photodiode Array Detector 2996 (Waters Corp., Milford, MA, U.S.A.) in *ca*. 6 × 10^-4^ M methanolic solution and log ε (the logarithm of molar absorption coefficient ε) was calculated for the absolute maximum λ_max_ of individual target compounds. Infrared spectra were recorded using KBr pellets on the spectrometer Nicolet Impact 400, for compounds **1**-**5**, and on the FT-IR spectrometer Nicolet 6700, for compounds **6**-**9**, (Nicolet - Thermo Scientific, USA). Wavenumbers are given in cm^-1^. All ^1^H-NMR and ^13^C-NMR spectra were recorded with a Varian Mercury-VxBB 300 spectrometer (299.95 MHz for ^1^H and 75.43 MHz for ^13^C; Varian Corp., Palo Alto, CA, USA). Chemical shifts were recorded as δ values in ppm and were indirectly referenced to tetramethylsilane (TMS) via the solvent signal (2.49 for ^1^H, 39.7 for ^13^C in DMSO-*d*_6_).

### 4.2. Synthesis

A mixture of an aldehyde or a ketone (0.009 mol) and rhodanine-3-acetic acid (0.009 mol) was dissolved in glacial acetic acid and equivalent amount of acetanhydride and sodium acetate were added. Then the reaction mixture was refluxed for 3 h. After cooling, the separated solid was filtered through a sintered filter, washed with distilled water (50 mL) and then with 50% ethanol (50 mL). The product was crystallized from glacial acetic acid.

*[(5Z)-(5-Benzylidene-4-oxo-2-thioxo-1,3-thiazolidin-3-yl)]acetic acid* (**1**). Yellow crystalline compound; Yield 66%; Mp 247-248 °C (240 °C, acetic acid [3]); Anal. Calcd. for C_12_H_9_NO_3_S_2_ (279.33): C 51.60%, H 3.25%, N 5.01%, S 22.96%; found: C 51.23%, H 3.08%, N 4.99%, S 20.75%; HPLC purity: 99.83%; UV (nm), λ_max_/log ε: 375.6/3.28; IR (KBr, cm^-1^): 3017 (CH), 1731 (C=O(OH)), 1707 (C=O), 759 (C=S); ^1^H-NMR (DMSO-*d*_6_), δ: 7.89 (1H, s, CH), 7.70-7.63 (2H, m, H2´, H6´), 7.60-7.51 (3H, m, H3´, H4´, H5´), 4.74 (2H, s, NCH_2_); ^13^C-NMR (DMSO-*d*_6_), δ: 193.5, 167.5, 166.6, 134.2, 133.0, 131.4, 131.0, 129.8, 122.0, 45.2.

*{(5Z)-[4-oxo-5-(pyridin-2-ylmethylidene)-2-thioxo-1,3-thiazolidin-3-yl]}acetic acid* (**2**). Yellow crystalline compound; Yield 76%; Mp 260-261 °C (253-254 °C, acetic acid [8]); Anal. Calcd. for C_11_H_8_N_2_O_3_S_2_ (280.32): C 47.13%, H 2.88%, N 9.99%, S 22.88%; found: C 46.75%, H 3.12%, N 9.81%, S 22.59%; HPLC purity: 98.95%; UV (nm), λ_max_/log ε: 384.4/3.29; IR (KBr, cm^-1^): 3042 (CH), 1740 (C=O(OH)), 1705 (C=O), 750 (C=S); ^1^H-NMR (DMSO-*d*_6_), δ: 8.81 (1H, d, *J* = 4.7 Hz, H6), 8.03-7.92 (2H, m, H4, H5), 7.90 (1H, s, CH), 7.50-7.43 (1H, m, H3), 4.73 (2H, s, NCH_2_); ^13^C-NMR (DMSO-*d*_6_), δ: 199.9, 167.6, 166.7, 151.2, 149.8, 138.0, 129.7, 128.7, 126.4, 124.6, 44.8.

*{(5Z)-[4-Oxo-5-(pyridin-3-ylmethylidene)-2-thioxo-1,3-thiazolidin-3-yl]}acetic acid* (**3**). Yellow crystalline compound; Yield 61%; Mp 257-258 °C (261-264 °C, ethanol-acetic acid [27]); Anal. Calcd. for C_11_H_8_N_2_O_3_S_2_ (280.32): C 47.13%, H 2.88%, N 9.99%, S 22.88%; found C 46.89%, H 3.15%, N 9.86%, S 23.17%; HPLC purity: 98.75%; UV (nm), λ_max_/log ε: 388.9/3.29; IR (KBr, cm^-1^): 1704 (C=O), 745 (C=S); ^1^H-NMR (DMSO-*d*_6_), δ: 8.90 (1H, d, *J* = 2.3 Hz, H2), 8.67 (1H, d, *J* = 4.7 Hz, H6), 8.02 (1H, d, *J* = 7.9 Hz, H4), 7.94 (1H, s, CH), 7.59 (1H, dd, *J* = 7.9 Hz, *J* = 4.7 Hz, H5), 4.75 (2H, s, NCH_2_); ^13^C-NMR (DMSO-*d*_6_), δ: 193.1, 167.5, 166.4, 152.2, 151.4, 136.9, 130.9, 129.2, 124.6, 124.3, 45.3.

*{(5Z)-[4-Oxo-5-(pyridin-4-ylmethylidene)-2-thioxo-1,3-thiazolidin-3-yl]}acetic acid* (**4**). Yellow crystalline compound; Yield 80%; Mp 277-280 °C (263-266 °C, ethanol-acetic acid [27]); Anal. Calcd. for C_11_H_8_N_2_O_3_S_2_ (280.32): C 47.13%, H 2.88%, N 9.99%, S 22.88%; found: C 45.07%, H 3.32%, N 8.95%, S 22.42%; HPLC purity: 97.95%; UV (nm), λ_max_/log ε: 389.1/3.29; IR (KBr, cm^-1^): 3037 (CH), 1797 (C=O(OH)), 1708 (C=O), 754 (C=S); ^1^H-NMR (DMSO-*d*_6_), δ: 8.78-8.72 (2H, m, H2, H6), 7.86 (1H, s, CH), 7.63-7.56 (2H, m, H3, H5), 4.75 (2H, s, NCH_2_); ^13^C-NMR (DMSO-*d*_6_), δ: 193.0, 167.4, 166.3, 151.0, 139.9 130.9, 127.1, 124.0, 45.3.

*{(5Z)-[4-Oxo-5-(pyrazin-2-ylmethylidene)-2-thioxo-1,3-thiazolidin-3-yl]}acetic acid* (**5**). Yellow crystalline compound; Yield 71%; Mp 266-268 °C; Anal. Calcd. for C_10_H_7_N_3_O_3_S_2_ (281.31): C 42.70%, H 2.51%, N 14.94%, S 22.80%; found: C 42.57%, H 2.83%, N 14.80%, S 25.46%; HPLC purity: 97.85%; UV (nm), λ_max_/log ε: 389.2/3.29; IR (KBr, cm^-1^): 3051 (CH), 1737 (C=O(OH)), 1709 (C=O), 740 (C=S); ^1^H-NMR (DMSO-*d*_6_), δ: 9.14 (1H, s, H3), 8.87 (1H, s, H5), 8.67 (1H, s, H6), 7.94 (1H, s, CH), 4.57 (2H, s, NCH_2_); ^13^C-NMR (DMSO-*d*_6_), δ: 198.4, 167.4, 166.7, 148.8, 147.4, 144.7, 129.1, 125.6, 46.2.

*[(5Z)-{4-Oxo-5-[1-(pyrazin-2-ylethylidene)]-2-thioxo-1,3-thiazolidin-3-yl}]acetic acid* (**6**). Dark orange solid; Yield 75%; Mp 261-263 °C; Anal. Calcd. for C_11_H_9_N_3_O_3_S_2_ (295.34): C 44.74%, H 3.07%, N 14.23%, S 21.71%; found: C 44.41%, H 3.20%, N 14.05%, S 21.16%; HPLC purity: 98.95%; UV (nm), λ_max_/log ε: 388.0/3.19; IR (KBr, cm^-1^): 3412 (C-OH), 3042 (CH), 1716 (C=O), 784 (C=S); ^1^H-NMR (DMSO-*d*_6_), δ: 3.37 (1H, bs, OH), 9.33 (1H, d, *J* =1.7 Hz, H3), 8.92-8.89 (1H, m, H5), 8.74 (1H, d, *J* =2.5 Hz, H6), 4.71 (2H, s, NCH_2_), 2.92 (3H, s, CH_3_); ^13^C-NMR (DMSO-*d*_6_), δ: 199.5, 167.7, 166.5, 149.4, 145.7, 144.7, 142.3, 139.5, 123.2, 44.7, 15.5.

*[(5Z)-{5-[1-(5-Butylpyrazin-2-yl)ethylidene]-4-oxo-2-thioxo-1,3-thiazolidine-3-yl}]acetic acid* (**7**). Yellow crystalline compound; Yield 77%; Mp 213-215 °C; Anal. Calcd. for C_15_H_17_N_3_O_3_S_2_ (351.45): C 51.26%, H 4.88%, N 11.96%, S 18.25%; found: C 50.84%, H 4.89%, N 11.96%, S 17.88%; HPLC purity: 99.03%; UV (nm), λ_max_/log ε: 388.0/3.29; IR (KBr, cm^-1^): 3398 (C-OH), 1706 (C=O), 758 (C=S); ^1^H-NMR (DMSO-*d*_6_), δ: 9.22 (1H, d, *J* = 1.4 Hz, H3), 8.81 (1H, d, *J* = 1.4 Hz, H6), 4.71 (2H, s, NCH_2_), 2.90 (3H, s, CH_3_), 2.86 (2H, t, *J* = 7.5 Hz, CH_2_), 1.78-1.63 (2H, m, CH_2_), 1.41-1.25 (2H, m, CH_2_), 0.90 (3H, t, *J* = 7.5 Hz, CH_3_); ^13^C-NMR (DMSO-*d*_6_), δ: 199.5, 167.7, 166.5, 157.7, 146.8, 144.8, 141.7, 139.9, 122.0, 44.7, 34.4, 30.8, 22.0, 15.5, 13.9.

*[(5Z)-{5-[1-(5-tert-Butylpyrazin-2-yl)ethylidene]-4-oxo-2-thioxo-1,3-thiazolidin-3-yl}]acetic acid* (**8**). Yellow crystalline compound; Yield 63%; Mp 254-261 °C (decomp.); Anal. Calcd. for C_15_H_17_N_3_O_3_S_2_ (351.44): C 51.26%, H 4.88%, N 11.96%, S 18.25%; found: C 50.85%, H 5.10%, N 12.12%, S 18.32%; HPLC purity: 98.42%; UV (nm), λ_max_/log ε: 388.0/3.16; IR (KBr, cm^-1^): 3392 (C-OH), 1703 (C=O), 758 (C=S); ^1^H-NMR (DMSO-*d*_6_), δ: 13.37 (1H, bs, OH), 9.26 (1H, d, *J* = 1.4 Hz, H3), 9.03 (1H, d, *J* = 1.4 Hz, H6), 4.71 (2H, s, NCH_2_), 2.92 (3H, s, CH_3_), 1.39 (9H, s, CH_3_); ^13^C-NMR (DMSO-*d*_6_), δ: 199.6, 167.8, 166.5, 163.9, 146.5, 144.1, 139.7, 139.1, 122.2, 44.7, 36.9, 29.6, 15.5.

*[(5Z)-{5-[1-(5-Benzylpyrazin-2-yl)ethylidene]-4-oxo-2-thioxo-1,3-thiazolidin-3-yl}]acetic acid* (**9**). Yellow crystalline compound; Yield 31%; Mp 224-225 °C; Anal. Calcd. for C_18_H_15_N_3_O_3_S_2_ (385.47): C 56.09%, H 3.92%, N 10.90%, S 16.64%; found: C 55.29%, H 3.96%, N 10.86%, S 16.46%; HPLC purity: 99.62%; UV (nm), λ_max_/log ε: 386.8/3.32; IR (KBr, cm^-1^): 3436 (C-OH), 1729 (C=O(OH)), 1697 (C=O), 754 (C=S); ^1^H-NMR (DMSO-*d*_6_), δ: 9.22 (1H, d, *J* = 1.4 Hz, H3), 8.88 (1H, d, *J* = 1.4 Hz, H6), 7.36-7.18 (5H, m, Ar), 4.70 (2H, s, NCH_2_), 4.23 (2H, s, CH_2_), 2.89 (3H, s, CH_3_); ^13^C-NMR (DMSO-*d*_6_), δ: 199.4, 167.7, 166.5, 156.3, 147.1, 145.0, 141.8, 139.7, 138.5, 129.3, 128.8, 126.8, 122.3, 44.8, 40.9, 15.5.

### 4.3. Lipophilicity HPLC determination (capacity factor k/calculated log k)

The HPLC separation module Waters Alliance 2695 XE and Waters Photodiode Array Detector 2996 (Waters Corp., Milford, MA, U.S.A.) were used. The chromatographic column Symmetry^®^ C_18_ 5 μm, 4.6 × 250 mm, Part No. WAT054275, (Waters Corp.) was used. The HPLC separation process was monitored by Millennium32^®^ Chromatography Manager Software, Waters 2004 (Waters Corp.). A mixture of MeOH p.a. (70.0%) and H_2_O-HPLC – Mili-Q Grade (30.0%) was used as a mobile phase. The total flow of the column was 0.9 mL/min, injection 30 μL, column temperature 30 °C and sample temperature 10 °C. The detection wavelength 210 nm was chosen. The KI methanolic solution was used for the dead time (t_D_) determination. Retention times (t_R_) were measured in minutes.

The capacity factors *k* were calculated using the Millennium32^®^ Chromatography Manager Software according to formula *k* = (t_R_ - t_D_) / t_D_, where t_R_ is the retention time of the solute, whereas t_D_ denotes the dead time obtained via an unretained analyte. Log *k*, calculated from the capacity factor *k*, is used as the lipophilicity index converted to log *P* scale. The log *k* values of the individual compounds are shown in Table 1.

### 4.4. In vitro evaluation of antifungal activity

All strains were subcultured on Sabouraud dextrose agar (SDA, Difco) and maintained on the same medium at 4 °C. Prior to testing, each strain was passaged onto SDA and fungal inocula were prepared by suspending yeasts or conidia or sporangiospores in sterile 0.85% saline. The cell density was adjusted, using the Bürker’s chamber, to yield a stock suspension of (1.0 ± 0.2) × 105 CFU/mL. The final inoculum was made by 1:20 dilution of the stock suspension with the test medium. The compounds were dissolved in dimethyl sulfoxide (DMSO) and antifungal activity was determined in the tissue culture medium RPMI 1640 (Sevapharma, Prague, Czech Republic) buffered to pH 7.0 with 0.165 M 3-morpholinopropane-1-sulfonic acid (Sigma-Aldrich). Controls consisted of medium and DMSO alone. The final concentration of DMSO in the test medium did not exceed 1% (v/v) of the total solution composition. The minimum inhibitory concentration (MIC), defined as 80% inhibition of fungal growth compared to control, were determined after 24 and 48 h of static incubation at 35 °C. In the case of *T. mentagrophytes* the MICs were recorded after 72 and 120 h. Fluconazole (Pfizer, New York, NY, USA) was used as reference antifungal drugs. The antifungal evaluation results are showed in Table 2.
